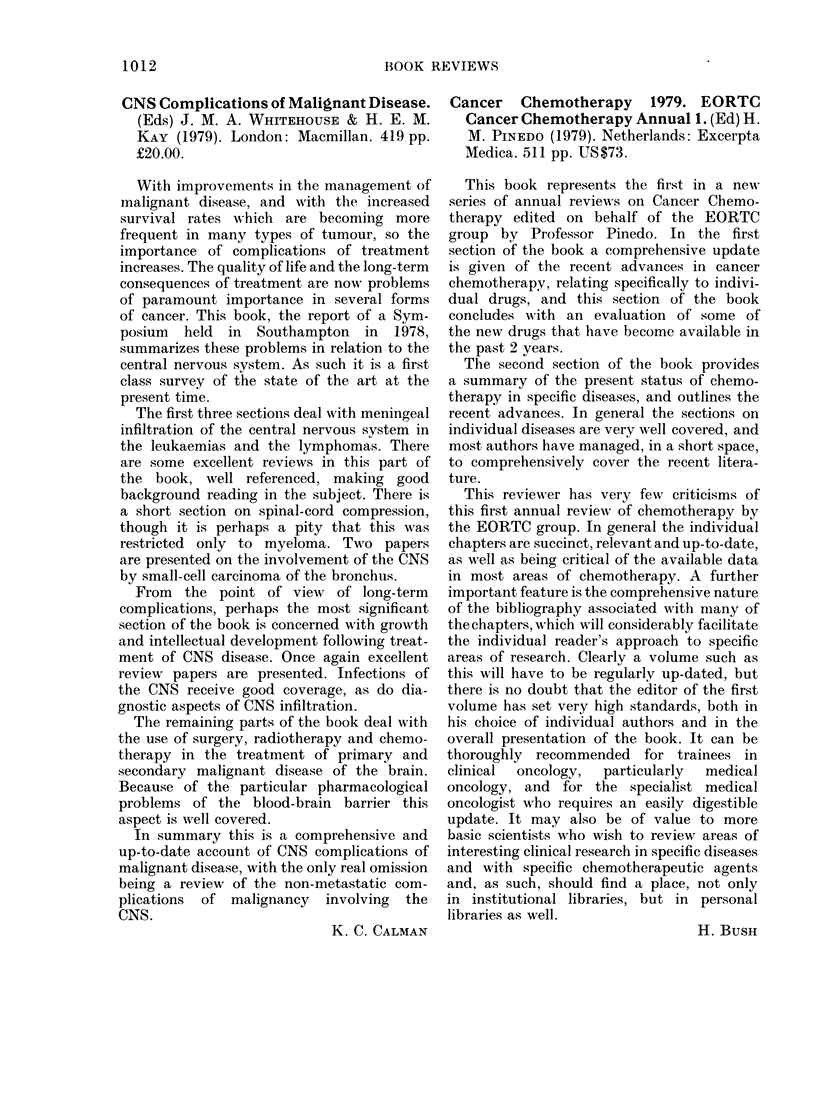# CNS Complications of Malignant Disease

**Published:** 1980-06

**Authors:** K. C. Calman


**CNS Complications of Malignant Disease.** (Eds) J. M. A. Whitehouse & H. E. M. Kay (1979). London: Macmillan. 419 pp. £20.00.

With improvements in the management of malignant disease, and with the increased survival rates which are becoming more frequent in many types of tumour, so the importance of complications of treatment increases. The quality of life and the long-term consequences of treatment are now problems of paramount importance in several forms of cancer. This book, the report of a Symposium held in Southampton in 1978, summarizes these problems in relation to the central nervous system. As such it is a first class survey of the state of the art at the present time.

The first three sections deal with meningeal infiltration of the central nervous system in the leukaemias and the lymphomas. There are some excellent reviews in this part of the book, well referenced, making good background reading in the subject. There is a short section on spinal-cord compression, though it is perhaps a pity that this was restricted only to myeloma. Two papers are presented on the involvement of the CNS by small-cell carcinoma of the bronchus.

From the point of view of long-term complications, perhaps the most significant section of the book is concerned with growth and intellectual development following treatment of CNS disease. Once again excellent review papers are presented. Infections of the CNS receive good coverage, as do diagnostic aspects of CNS infiltration.

The remaining parts of the book deal with the use of surgery, radiotherapy and chemotherapy in the treatment of primary and secondary malignant disease of the brain. Because of the particular pharmacological problems of the blood-brain barrier this aspect is well covered.

In summary this is a comprehensive and up-to-date account of CNS complications of malignant disease, with the only real omission being a review of the non-metastatic complications of malignancy involving the CNS.

K. C. Calman